# Overexpression of MN1 Confers Resistance to Chemotherapy, Accelerates Leukemia Onset, and Suppresses p53 and Bim Induction

**DOI:** 10.1371/journal.pone.0043185

**Published:** 2012-08-14

**Authors:** Timothy S. Pardee

**Affiliations:** 1 Wake Forest University Health Sciences, Department of Internal Medicine, Section on Hematology and Oncology, Winston-Salem, North Carolina, United States of America; 2 Wake Forest School of Medicine, Department of Cancer Biology, Comprehensive Cancer Center, Winston-Salem, North Carolina, United States of America; Institut national de la santé et de la recherche médicale (INSERM), France

## Abstract

**Background:**

The transcriptional co-activator MN1 confers a worse prognosis for patients with acute myeloid leukemia (AML) when highly expressed; however, the mechanisms involved are unknown. We sought to model the effects of high MN1 expression in AML models to explore the underlying mechanisms.

**Methodology/Principal Findings:**

We used cell lines and a genetically defined mouse model of AML to examine the effects of MN1 overexpression on prognosis and response to cytarabine and doxorubicin *in vitro* and *in vivo*. Murine AML that was engineered to overexpress MN1 became more aggressive *in vivo*, leading to shortened survival in both treated and control groups. *In vitro* murine AML cells that overexpressed MN1 became resistant to treatment with cytarabine and highly resistant to doxorubicin. This resistant phenotype was also seen *in vivo*, where treatment with the combination of cytarabine and doxorubicin selected for cells expressing MN1. When therapy-induced DNA damage levels were assessed by γH2AX foci, no reduction was seen in MN1 expressing cells arguing against a drug efflux mechanism. Despite no reduction in DNA damage, MN1-expressing cells showed less apoptosis as assessed by annexin V and propidium iodide staining. Following treatment, p53 and BIM induction were markedly reduced in cells expressing MN1. Pharmacologic inhibition of the p53 E3 ligase MDM2 resulted in increased p53 levels and improved response to doxorubicin in vitro.

**Conclusions/Significance:**

MN1 overexpression accelerates an already aggressive leukemia, confers resistance to chemotherapy, and suppresses p53 and BIM induction, resulting in decreased apoptosis. This provides a mechanistic explanation of the poor prognosis observed with high MN1 expression and suggests that therapies directed at increasing p53 function may be useful for these patients.

## Introduction

Acute myeloid leukemia (AML) is an aggressive malignancy of the myeloid lineage that leads to progressive marrow failure and death. It affects approximately 13,000 Americans every year, leading to over 9,000 deaths [1]. Despite decades of research, therapies for most patients have remained unchanged and outcomes are poor [2]. AML is a very genetically heterogenous disease; this heterogeneity is thought to underlie the diverse responses to therapy. Cytogenetics are the single most important prognostic marker in AML. Patients can be placed into poor, intermediate, and good risk categories depending on the presence of different chromosomal rearrangements [3], [4], [5], [6]. Most patients with AML have a normal karyotype, placing them in the intermediate category; however, within this category, patients have very diverse outcomes. In these patients prognosis is affected by the presence of mutations or overexpression of certain genes [7], [8].

The meningioma 1 (*MN1*) gene adversely affects prognosis when highly expressed in AML patients [9], as reported in multiple independent cohorts and in both younger and older patients [10], [11]. MN1 is thought to be a transcriptional co-activator and, through its interactions with p300 and RAC3, to be involved in regulating transcriptional targets of the retinoic acid and vitamin D receptors [12], [13], [14], [15]. Overexpression of MN1 generates AML in murine bone marrow experiments and predicts resistance to all-trans retinoic acid (ATRA) therapy in elderly AML patients [16]. MN1 overexpression also cooperates with MLL-ENL and CBFβ-SMMHC in the generation of murine AML [17], [18].

Despite these studies, the mechanisms by which MN1 overexpression confers an adverse prognosis remain unclear. To address this question, we have altered a genetically defined murine AML model and human cell lines to express high levels of MN1, and determined the effects on prognosis and response to standard therapy.

## Materials and Methods

### Ethics Statement

All animal studies were approved by the Wake Forest University Health Sciences IACUC committee.

### Retroviral Constructs

All retroviruses were constructed in the MSCV backbone (Clontech, Mountain View, CA). MN1-overexpressing vector was generated using the gateway cloning system (Invitrogen, Grand Island, NY). The destination vector (pMGWIG, a kind gift of Dr Uli Bialucha) was created with attR1 and R2 sites placed upstream of an IRES GFP in MSCV. MN1 full-length ORF in pENTR223.1 was purchased from Open Biosystems. The resulting MN1 IRES GFP vector was confirmed by sequencing. The GFP-only vector contained the full-length GFP ORF only. Cloning strategies are available on request.

### Cell Culture and Viability Assays

OCI-AML3 cells [19] were a kind gift of Dr Mark Minden at University of Toronto. Molm-13 cells [20] were purchased from the DSMZ. All human cell lines were maintained in RPMI media (GIBCO, Carlsbad, CA) supplemented with 10% FBS, penicillin, and streptomycin. Cells were grown at 37°C with 5% CO_2_. Viability assays were done using the Cell Titer-Glo assay (Promega, Madison, WI) according to the manufacturer’s protocol, or by Trypan blue exclusion assay using the Countess cell counting system (Invitrogen, Carlsbad, CA). All murine cells were derived from fetal liver cells infected with MLL-ENL alone or with Flt3-ITD-expressing vectors (M1p5, MF3 and MFL2) [21]. Murine cells were maintained in stem cell media (40% DMEM, 40% IMDM, 20% FBS, supplemented with murine SCF to 10 ng/ml, murine IL6 to 2 ng/ml, and murine IL3 to 0.4 ng/ml).

### Competition Assays

Two days after infection or thereafter, leukemia cells were split into replicate wells of ∼50,000 cells in 24-well plates. After 72-hour treatments, the GFP-positive percentage was quantified in the live cell population by using a FACSCalibur flow cytometer (BD Biosciences, San Jose, CA). For cultures where the GFP+ population was in excess of 50%, non-infected co-cultured cells were added to achieve a final GFP+ population of ∼20%.

### Quantitative PCR Assays

Cells were treated as indicated and total RNA harvested using RNeasy mini kits including treatment with DNAse, as per the manufacturer’s protocol (Qiagen, Valencia, CA). Initial RNAs isolated in this manner were assessed for DNA contamination with no reverse transcriptase controls. Following isolation RNA was analyzed and quantified by spectrophometric analysis and stored at −80 degrees. 1 µg of total RNA was converted to cDNA using the iScript cDNA synthesis kit in 20 µl reactions (Bio-Rad, Hercules, CA). Following addition of all reagents, samples were placed at 25 degrees for 5 minutes then heated to 42 degrees for 30 minutes then heated to 85 degrees for 5 minutes. QPCR was carried out on a CFX-96 QPCR machine using SsoFast EvaGreen Mastermix as per the manufacturer’s protocol (Bio-Rad, Hercules, CA). After an initial heating to 95 degrees for 30 seconds a two step protocol was used. 20 µl reactions were heated to 95 degrees for 10 seconds then cooled to 60 degrees for 30 seconds for 40 cycles followed by a melting curve from 65 to 95 degrees in 0.5 degree increments for 5 seconds. Product specificity was determined by standard melting curves and analyzed using Bio-Rad CFX manager software version 1.6.541.1028. Relative message levels were calculated using the ΔΔCt method and normalized to actin. Cells not treated served as controls. Primer sequences are available on request.

### Western Blots and Immunofluorescence

Samples were lysed in Laemmli buffer, separated by SDS-PAGE, and transferred to an Immobilon PVDF membrane (Millipore). Antibodies against p53 (IMX25, 1∶1000; Leica Microsystems), p21 (C-19,1∶500; Santa Cruz Biotechnologies), and actin (AC-15, 1∶5000; Abcam) were used. Immunofluorescence for phosphorylated γH2AX, cells were fixed with 4% NBF, permeabilized with PBS containing 0.2% Triton-X 100, and probed with anti-phosphoH2AX (1∶100, #2577; Cell Signalling Technologies) followed by donkey anti-rabbit Alexa 594 conjugated antibody (1∶500, A-21207; Invitrogen) and visualized via fluorescence microscopy. Greater than 90 nuclei were then scored per condition, per experiment, using the following scoring system: 0 = none, 1 = few, 2 = moderate, 3 = many.

### Transplantation and in vivo Treatment Studies

For treatment studies, primary leukemias were transplanted into 6- to 8-wk-old sublethally irradiated recipient C57Bl/6 mice (4 Gy) by tail-vein injection of 1×10^6^ viable cells. Sublethal irradiation was used to achieve a more uniform disease onset in recipient animals. Mice transplanted with luciferase-expressing leukemias were monitored by bioluminescent imaging on day 7 after transplantation. Bioluminescent imaging was performed using an IVIS100 imaging system (Caliper LifeSciences, Hopkinton, MA). Mice were injected intraperitoneally with 150 mg/kg D-luciferin (Caliper LifeSciences), anesthetized with isoflurane, and imaged for 2 min after a 5-min incubation following injection. Chemotherapy was initiated upon detection of clear signals. Mice were treated for five consecutive days every 24 h with intraperitoneal injections of 100 mg/kg cytarabine and 3 mg/kg doxorubicin (both from Bedford Laboratories, Bedford, OH). Control animals were observed only.

### In vivo Competition Assays

Ly5.1+ C57Bl6 6- to 8-wk-old sublethally irradiated recipient mice (4 Gy) were injected with 1×10^6^ blasts infected with MN1- or GFP-expressing virus. These mice are syngeneic with standard C57Bl6 mice except for the Ly5.1 allele, allowing us to distinguish between endogenous marrow cells and Ly5.2+ leukemia cells. On day 13, observation or treatments with doxorubicin and cytarabine as above were begun. After 5 days of treatment or observation, animals were sacrificed and bone marrow was harvested from both femurs. Tissues were stained with APC conjugated anti-Ly5.2 antibody (E Biosciences, San Diego, CA) and then analyzed by flow cytometry using a FACSCalibur flow cytometer (BD Bioscience, San Jose, CA).

### Statistical Methods

In all experiments with more than two means, an initial ANOVA analysis was used. If a statistically significantly result was obtained (p<0.05), individual means were compared by two-sided standard Student’s T test. For comparison of Kaplan-Meier curves, a log-rank test was used. All statistical analysis was performed using Graph Pad Prism 5 software (GraphPad Software Inc). All error bars represent the standard error.

## Results

**Figure 1:**
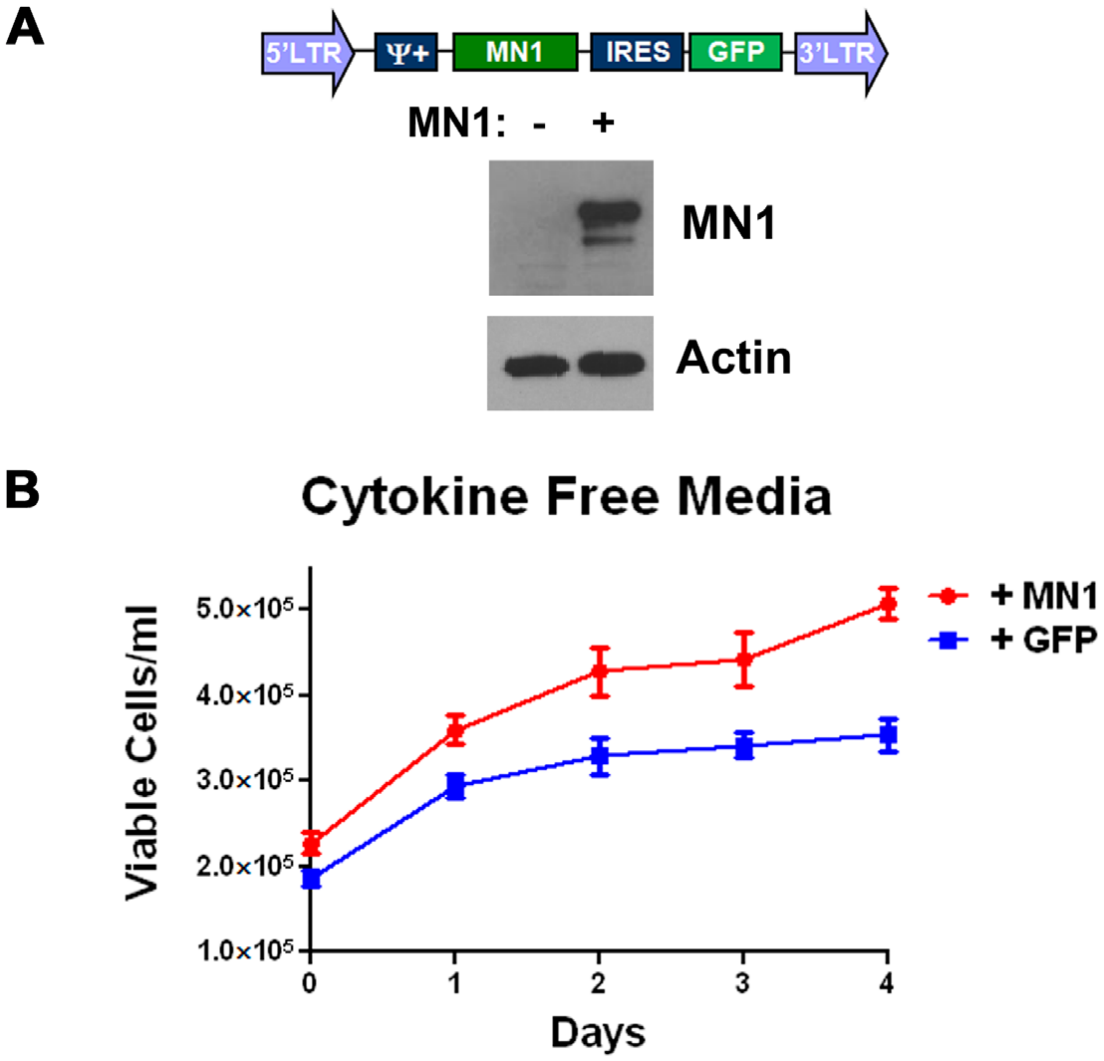
MN1 overexpression confers cytokine independence. A) Western blot for MN1. Retroviral vector expressing human MN1 in a bicistronic message with GFP shown in diagram was transfected into HeLa cells. After 48 hours, cells were lysed and blotted for MN1. B) Growth curves. The cytokine dependent MLL-ENL expressing murine AML cell line M1p5 was infected with either the MN1 expressing or GFP only expressing vector and grown in media without murine SCF, iL6 and iL3. Shown is the average of three independent experiments each done in triplicate. Error bars represent the standard error of measurement.

MN1 overexpression confers cytokine independence in a murine model of AML. To study the effects of MN1 overexpression, we used a genetically defined mosaic murine model of AML driven by MLL-ENL expression. We and others have previously shown that leukemias generated by this model can be cultured indefinitely in cytokine-supplemented media, infected with additional retroviruses, and can alter response to chemotherapy when expressing different oncogenes [21], [22]. The schema for AML generation is shown in Figure S1A. Since MN1 alters growth of AML cells [18] we overexpressed MN1 and assessed for cytokine dependence. The vector produced detectable full-length MN1 when transiently transfected into HeLa cells (Figure 1B). When we infected AML cells with this vector, they displayed a growth advantage in media without cytokine supplementation, unlike cells infected a GFP-expressing control retrovirus (Figure 1B). This was consistent with the loss of the GFP negative cells when partially infected populations were grown in media without cytokines (Figure S1B). These data demonstrate that functional MN1 is overexpressed in our system and can compensate for loss of cytokine growth signalling in an MLL-ENL driven murine model of AML.

**Figure 2:**
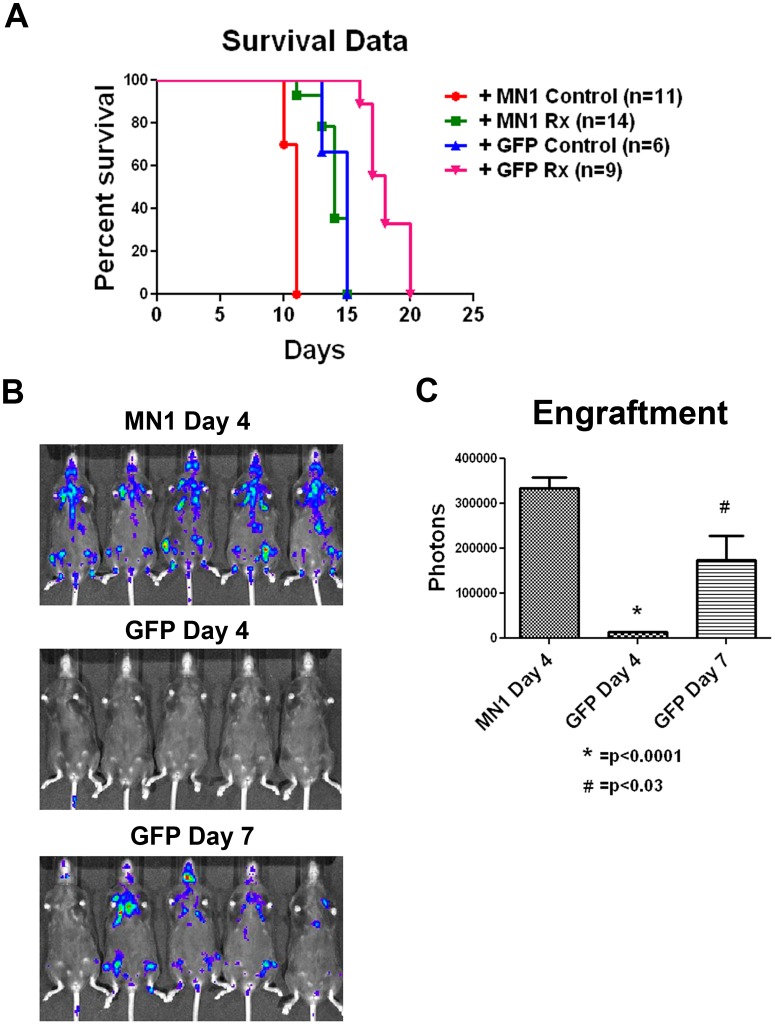
MN1 confers a worse survival in a genetically defined murine AML model. A) Kaplan-Meier survival curves. The indicated numbers of C57Bl/6 mice were injected with 1×10^6^ cells of GFP or MN1-expressing leukemia, treated or observed, and followed for survival. Rx = cytarabine at 100 mg/kg and doxorubicin at 3 mg/kg via IP injection daily for 5 days. Control = observation only. B) Bioluminescence imaging. Animals injected as in A were injected with 150 mg/kg D-luciferin and imaged on the indicated day. C) Quantitation of bioluminescence. Luminescence from animals in B was quantitated and graphed. Error bars represent the standard error of measurement. P value was calculated using a 2 tailed students T test.

MN1 overexpression impairs survival in a murine AML model. In order to assess the impact of MN1 overexpression in our model system, we infected luciferase-tagged, MLL-ENL- and Flt3 ITD-expressing MFL2 cells with the MN1 or control vector. Blasts were then injected into sublethally irradiated recipients. Once engraftment was established by bioluminescent signaling, animals were assigned to treatment or control groups. Treated animals were given doxorubicin at 3 mg/kg plus cytarabine at 100 mg/kg intraperitoneally for 5 days, and followed for survival. Both control and treated mice with MN1-expressing leukemia had a significantly shorter survival (p = 0.0006 for controls; and p<0.0001 for treated mice; Figure 2A). The accelerated engraftment was reproducible, with GFP expressing cells requiring 7 days to generate detectable bioluminescent signals compared to 4 days for MN1 expressing cells (Figure 2B). Even at day 7 when signals were quantitated, luminescence of GFP expressing cells was not equivalent to day 4 signals in MN1 cells (Figure 2C). This accelerated onset occurred despite the fact that the parental leukemia was already aggressive, with a median survival of 15 days. Areas of early engraftment also seemed to be affected by MN1. The GFP-only leukemia engrafted in the liver and spleen, while the MN1-expressing leukemia primarily engrafted in sternum, ribs, and pelvic bones (Figure 2B). To confirm and extend these results, we compared the survival of Ly5.1 C57Bl/6 mice injected with MFL2 cells with MN1 or control vector. Control mice had a median survival of 20 days versus 13 days for mice injected with MN1-expressing cells (p<0.0001, Figure S2). These data demonstrate that MN1 overexpression in this mouse model results in inferior survival consistent with the adverse prognosis seen in human patients.

**Figure 3:**
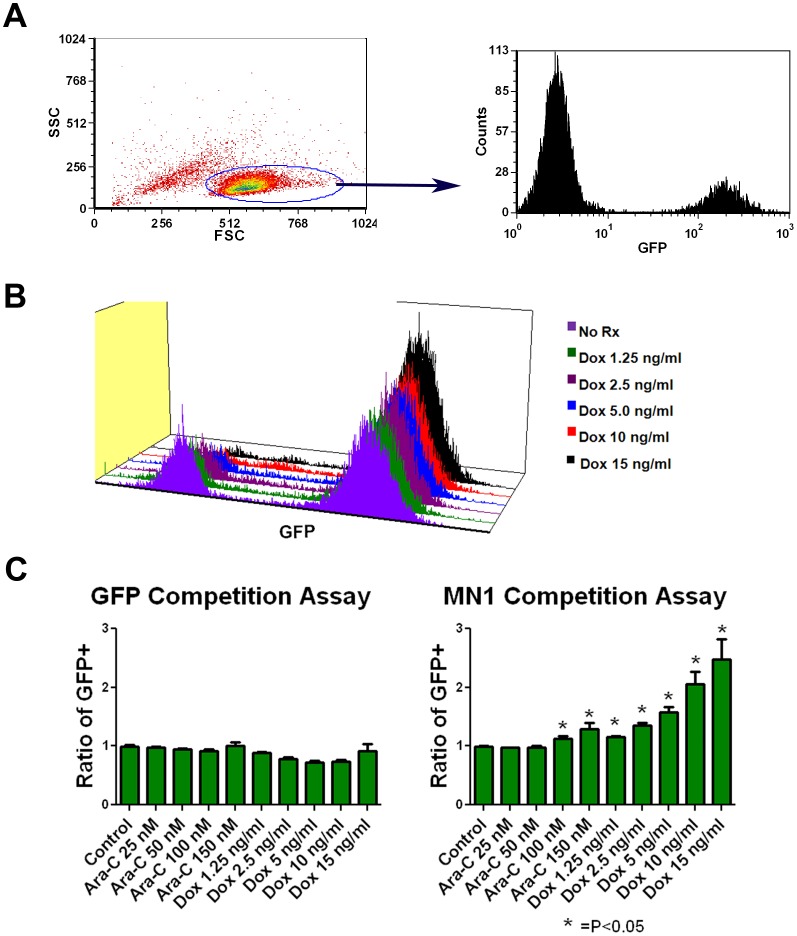
MN1 expression confers resistance to doxorubicin and cytarabine *in vitro*. A) Gating strategy. M1p5 cells were incubated with or without chemotherapy for 72 hours and then the viable population gated and assessed for GFP positivity. B) Flow cytometry histograms. Partially infected population of M1p5 cells were exposed to increasing amounts of doxorubicin as indicated for 72 hours and analyzed for GFP expression. Shown are histograms for GFP expression of the viable cell population from a representative experiment. C) Competition assays. Partially infected M1p5 cells were exposed to the indicated treatment for 72 hours. GFP-positive percentage in the viable population was determined and normalized to untreated controls. Shown is the average of three independent experiments each done in triplicate. Error bars represent the standard error of measurement. P value was calculated using a 2 tailed students T test.

MN1 confers resistance to chemotherapy *in vitro*. To further explore the mechanism of the adverse prognosis we determined the effect of MN1 expression on response to chemotherapy. We used cytarabine and an anthracycline, as they were the backbone of the treatment regimens patients received in the studies establishing the adverse impact of MN1 [9], [10], [11]. We used doxorubicin as the anthracycline to facilitate in vivo comparisons as daunorubicin is toxic to mice [23]. To assess the effects of MN1 on response, we employed the competition assay previously published in the same murine model [21]. In this assay the MLL-ENL expressing cell line M1p5 was partially infected with the MN1 vector and exposed to drug and then the viable fraction was gated and the GFP+ population quantitated (see gating strategy Figure 3A). When partially infected M1p5 cells were cultured with increasing amounts of doxorubicin for 72 hours, the viable population became selectively depleted of GFP-negative (and therefore MN1-negative) cells (Figure 3B). This result was highly reproducible. When the ratio of GFP+ cells was determined in three independent experiments, each done in triplicate, a significant and dose dependent enrichment was observed when MN1 expressing cells were exposed to doxorubicin (Figure 3C). MN1-expressing cells were also preferentially retained in the viable population when treated with cytarabine, although to a lesser extent (Figure 3C). In contrast, our GFP control vector did not confer an advantage in this assay, and there was no preferential retention of GFP vector cells with either doxorubicin or cytarabine exposure (Figure 3C). This was consistent with our recently published work demonstrating an increase in the IC50 of murine AML cells expressing MN1 for cytarabine, doxorubicin and to a lesser extent the novel fluoropyrimidine FdUMP [10] [24]. We and others have shown that the presence of the internal tandem duplication mutation of the FLT3 receptor tyrosine kinase (Flt3 ITD) confers resistance to anthracyclines [21], [25]. We sought to determine if MN1 overexpression could further increase resistance to anthracyclines in the presence of the Flt3 ITD. When we exposed the MLL-ENL and Flt3 ITD expressing cell line MF3 that was partially infected with the MN1 vector to doxorubicin, we again saw a selective enrichment of MN1-expressing cells (Figure S3A). As was the case with the M1p5 cells this result was highly reproducible and when the GFP+ ratio was determined in three independent experiments involving both MF3 and MFL2 cell lines, exposure to doxorubicin or cytarabine resulted in a significant enrichment in MN1 expressing cells (Figure S3B). When we exposed pure populations of MF3 cells expressing either GFP or MN1 to a titration of cytarabine or doxorubicin we saw a profound increase in resistance to doxorubicin and a significant resistance to cytarabine (Figure S3C). These data demonstrate that MN1 expression can confer resistance to both cytarabine and doxorubicin in murine AML cell lines with and without Flt3 ITD expression.

**Figure 4:**
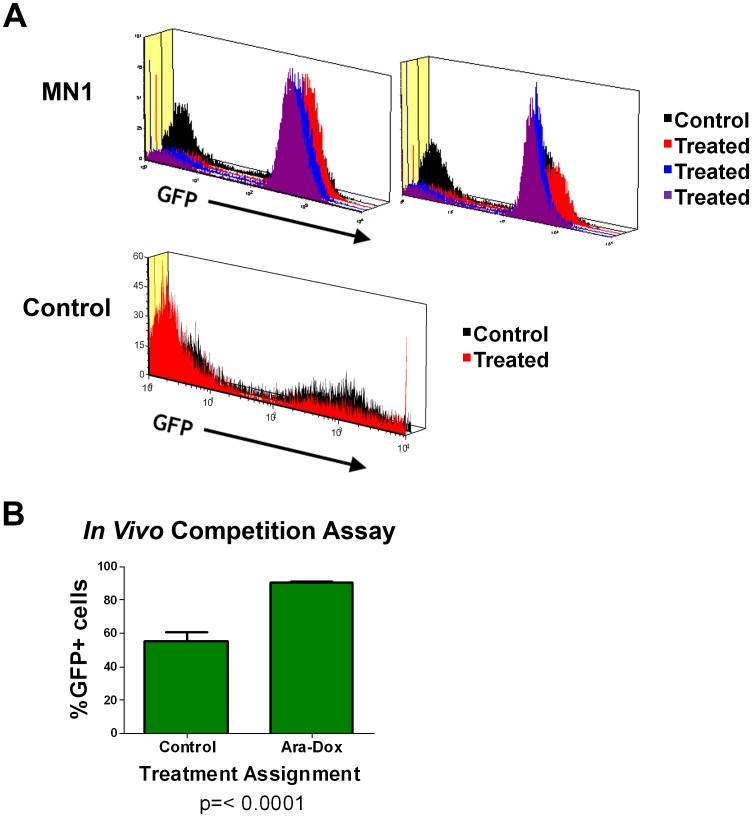
MN1 confers resistance to cytarabine and doxorubicin *in vivo*. A) Flow cytometry histogram. Bone marrow cells were harvested from both femurs of Ly5.1+ recipients of the MLL-ENL and Flt3 ITD expressing cell line MF3 infected with either MN1 or GFP expressing vectors following treatment or observation. Mice were treated as in figure 2. Cells were stained for Ly5.2 expression and GFP-positive percentages determined as outlined in the gating strategy (Figure S4). Shown are histograms for GFP expression of the Ly5.2+ population for each individual mouse. Each group of histograms represents mice from an independent experiment. B) GFP-positive percentages in the Ly5.2+ cell population from the experiment in panel A. Error bars represent the standard error of measurement. P value was calculated using a 2 tailed students T test.

MN1 expression confers resistance to therapy in vivo. Response of AML cells to therapy can be influenced by the microenvironment present in the marrow [26]. To assess the effect of MN1 expression in a setting that would incorporate these important interactions we performed an *in vivo* competition assay. In this assay, Ly5.1+ C57Bl/6 mice were injected with a mixed population of Ly5.2+ MF3 cells and treated with cytarabine plus doxorubicin for 5 days. Mice were then sacrificed and marrow cells collected, stained for Ly5.2, and the percentage of GFP+ cells was determined (see schema in Figure S4A and gating strategy in S4B). In two separate experiments, GFP-negative cells were selectively depleted in the marrow of treated mice compared to untreated controls (Figure 4A). In contrast, when animals were injected with MF3 cells infected with control vector, the GFP population was not enriched with treatment (Figure 4A). This enrichment of MN1-expressing cells was highly statistically significant (Figure 4B). These data suggest that MN1 expression is selected for during treatment, and that MN1 confers an advantage to cells under these conditions *in vivo*.

**Figure 5:**
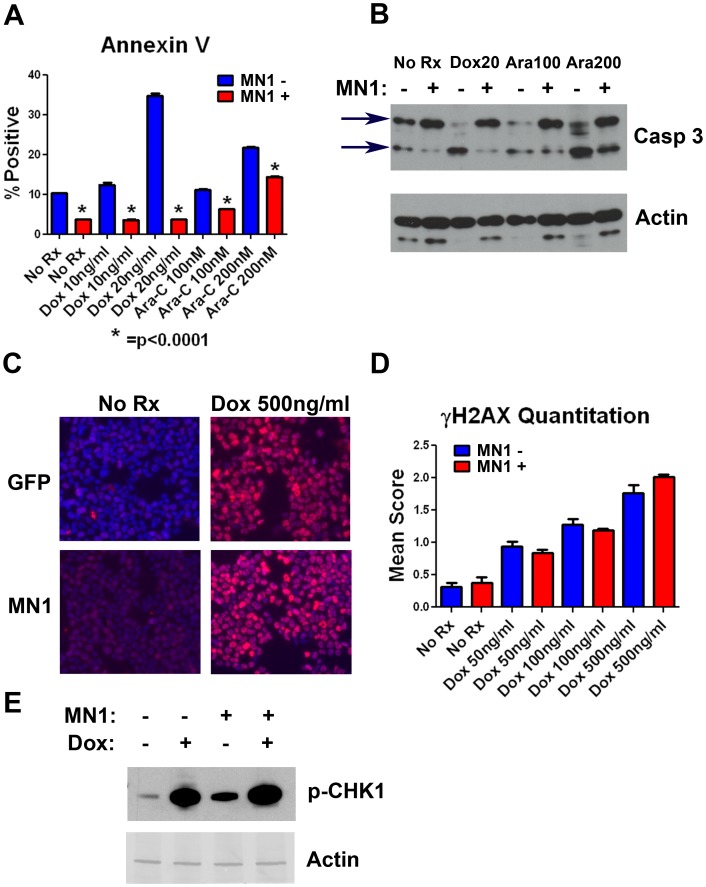
MN1 suppresses apoptosis but not DNA damage following therapy. A) Annexin V staining. MF3 cells expressing either MN1 or GFP were incubated for 24 hours in the indicated treatments. Following treatment, cells were stained with APC labeled annexin V and analyzed by flow cytometry. B) Caspase 3 Western blot. M1p5 cells expressing either MN1 or GFP were treated as indicated for 24 hours, lysed and blotted for caspase 3. Arrows indicate intact and cleaved caspase. C) γH2AX immunofluorescence staining. M1p5 cells were incubated with the indicated amount of doxorubicin for 4 hours, fixed, permeabilized, and stained with anti- γH2AX antibody. D) γH2AX quantitation. Shown is average γH2AX score for cells incubated with the indicated amount of doxorubicin. Nuclei were scored as explained in the methods. Shown is the average of 3 independent experiments each done in a different murine cell line (M1p5, MF3 and MFL2). MN1 vs GFP scores for each treatment were compared by two tailed students T test and were not significant. Error bars represent the standard error of measurement. E) Western blot. Phospho-CHK-1 levels were assessed by Western blot after 4 hour exposure to 500 ng/ml doxorubicin where indicated (+Dox). Actin served as a loading control.

MN1 decreases apoptosis but does not decrease the amount of DNA damage induced by doxorubicin. To better understand this therapeutic resistance, we determined the apoptotic response of cells with MN1 or control vector after exposure to cytarabine or doxorubicin. Control cells displayed a robust and dose-dependent increase in annexin V staining following treatment with either drug, whereas MN1-expressing cells had a marked reduction in annexin V staining, indicating a diminished apoptotic response to therapy (Figure 5A). To confirm and extend this result we performed annexin V and propidium iodide staining of cells expressing GFP or MN1. As in our annexin V studies MN1 over expression resulted in reduced induction of apoptosis (Figure S5A). This result was reproducible in separate experiments with independent cell lines (Figure S5B and data not shown). To further confirm this result using an independent assay we assessed caspase 3 cleavage by western blot in cells expressing GFP or MN1 after exposure to doxorubicin or cytarabine. Consistent with our flow cytometry data the MN1 expressing cells had reduced caspase 3 cleavage following exposure to drug (Figure 5B). These data demonstrate that over expression of MN1 results in reduced apoptotic response following exposure to cytarabine or doxorubicin in a murine model of AML.

Previous work has shown an association between MN1 and increased expression of MDR1 [10]; thus, we sought to determine if drug efflux played a significant role in the observed resistance. As the anthracyclines are known substrates of MDR1, we assessed the amount of γH2AX foci following treatment with doxorubicin between MN1 and GFP expressing cells. Nuclei were scored for the number of foci they contained, and mean scores were calculated and compared. γH2AX levels in MN1 expressing cells were not significantly different from GFP cells arguing against drug efflux as the primary mechanism of the observed resistance (Figure 5C and D). We also assessed phosphorylation of CHK-1, a kinase further downstream in the DNA damage response, following doxorubicin treatment. Consistent with the previous result, CHK-1 phosphorylation was not diminished in MN1-expressing cells (Figure 5E). Taken together, the evidence suggests that MN1-expressing cells are resistant to DNA damage-induced apoptosis.

**Figure 6:**
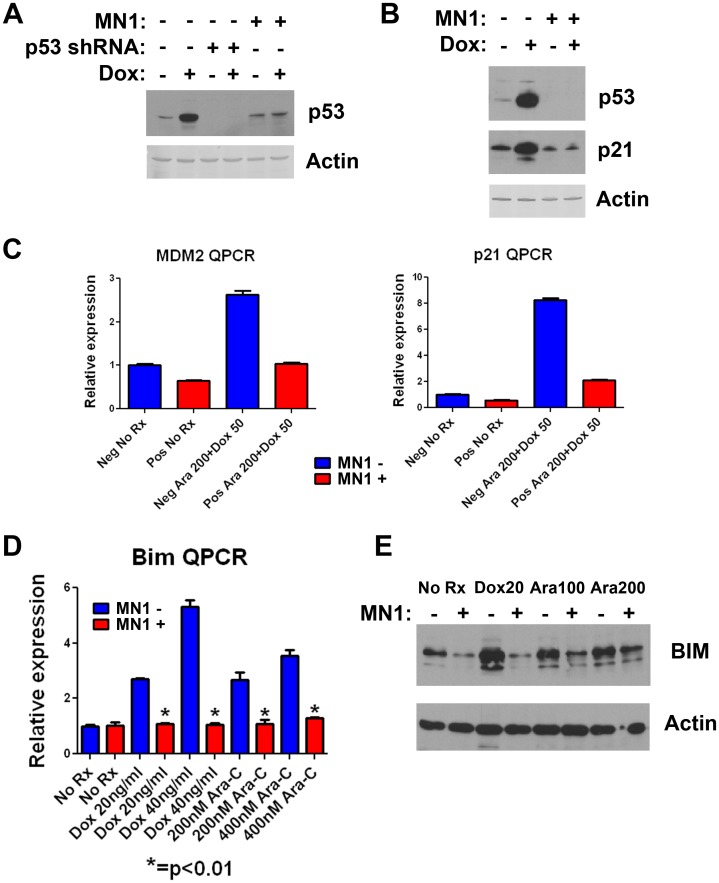
MN1 suppresses p53 and BIM induction following treatment. A) Western blot. M1p5 cells expressing MN1 or GFP were exposed to 500 ng/ml doxorubicin for 4 hours as indicated, lysed, and blotted for p53. p53 shRNA denotes M1p5 cells expressing an shRNA targeting p53. B) Western Blot. MF3 cells expressing MN1 or GFP were exposed to doxorubicin as in A, lysed, and blotted for p53 and p21. C) Quantitative RT-PCR assays. M1p5 cells expressing either MN1 or GFP were incubated with 50 ng/ml doxorubicin and 200 nM cytarabine as indicated for 24 hours. Cells were then lysed, total RNA harvested, and RT-QPCR done to determine the relative message levels of the p53 target genes MDM2 and p21. Message levels were set to 1 in untreated GPF- expressing cells. C) Quantitative RT-PCR assays. MF3 cells with or without MN1 overexpression were incubated with the indicated treatment for 24 hours. RT-QPCR was done to determine the relative message levels of BIM. Message levels were set to 1 in untreated GPF-expressing cells. E) Western Blot. M1p5 cells expressing MN1 or GFP were incubated with the indicated therapy for 24 hours, lysed and blotted for BIM protein. Dox20 = doxorubicin 20 ng/ml, Ara100 = cytarabine 100 nM, Ara200 = cytarabine 200 nM.

MN1 expression diminishes induction of p53 and BIM following exposure to chemotherapy. To better understand the decrease in apoptosis following therapy, we assessed the effect of MN1 expression on p53 induction. We chose to assess the p53 response, as we and others have demonstrated the central role of p53 in response to chemotherapy [21], [27], [28], [29]. Murine blasts with GFP or MN1 vectors were exposed to doxorubicin and p53 protein levels determined by western blot. Induction of p53 was markedly reduced in cells overexpressing MN1 (Figure 6A). This was also observed in a Flt3 ITD-expressing cell line (Figure 6B). To confirm and extend this result, we also assessed the induction of two canonical p53 targets, p21 and MDM2. Following treatment, p21 levels (measured by Western blots) and message level (measured by quantitative PCR) were markedly reduced in cells overexpressing MN1 compared to controls (Figure 6B and C). Likewise, MDM2 message levels were also reduced following treatment (Figure 6C). We then assessed sensitivity to the small molecule MDM2 antagonist Nutlin-3. Nutlin-3 increases p53 cellular levels by interfering with p53 binding to its E3 ubiquitin ligase MDM2 [30]. If MN1 suppresses p53, then MN1-overexpressing cells should display increased resistance to Nutlin-3. When MN1- or GFP-expressing murine AML cells were exposed to Nutlin-3, MN1-expressing cells displayed increased resistance, with an IC_50_ value nearly twice that of GFP-expressing cells (3.34 µM [95% CI 3.20 to 3.48] vs 5.80 µM [95% CI 5.34 to 6.30]; Figure S6A). This effect was seen in independent experiments and consistent across multiple cell lines. When p53 induction was assessed following treatment with Nutlin-3, MN1-expressing cells showed a diminished p53 response compared to GFP controls (Figure S6B). These data demonstrate that MN1 can decrease p53 induction following diverse stimuli including DNA damage and MDM2 inhibition.

In addition to p53, the proapoptotic BH3-only family member *BIM* can contribute to induction of apoptosis following therapy [31], [32]. We determined the effect of MN1 overexpression on induction of *BIM* mRNA using quantitative PCR. *BIM* message induction was significantly decreased following treatment with either doxorubicin or cytarabine (Figure 6D). This observation was reproducible in both MLL-ENL only and MLL-ENL- and Flt3 ITD-expressing AML cells (Data not shown). Western blot analysis confirmed that MN1 expression inhibited BIM induction (Figure 6E). Taken together, these data suggest that the resistance observed in MN1-overexpressing cells occurs via reduced apoptosis, likely as a consequence of suppression of p53 and BIM signaling.

Nutlin 3 increases the sensitivity of MN1 over expressing cells to doxorubicin. Although MN1 expressing cells were resistant to nutlin 3 when compared GFP expressor it was still capable of increasing the levels of p53 (Figure 6SB). Given this we sought to determine if exposure of cells to the combination of nutlin 3 and doxorubicin would decrease the MN1 mediated resistance. As expected when treated with the combination we saw a reduction in viability in the MN1 expressing cells when compared to those treated with doxorubicin only. However, even in the presence of nutlin 3 the MN1 expressing cells still had increased resistance when compared to GFP expressing cells indicating that nutlin 3 at the concentration used could only partially reverse MN1 mediated resistance (Figure S7). None the less these data would suggest that the administration of p53 agonists in combination with standard chemotherapy may be of benefit to patients with high MN1 levels.

**Figure 7:**
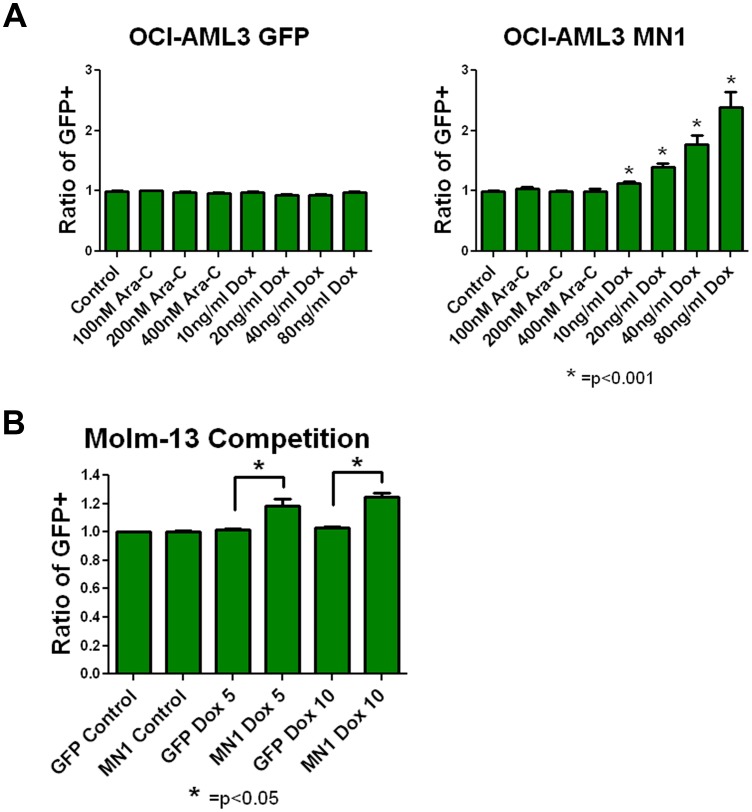
MN1 confers resistance to doxorubicin in human AML cells. A) Competition assays as in Figure 3C using OCI-AML3 cells. Cells were incubated with the indicated therapy for 72 hours, and percentage of GFP-positive cells normalized to untreated controls. Shown is the average of three independent experiments each done in triplicate. Error bars represent the standard error of measurement. P value was calculated using a 2 tailed students T test. B) Competition assays as in panel A, using Molm-13 cells. Shown is the average of two independent experiments each done in triplicate. Error bars represent the standard error of measurement. P value was calculated using a 2 tailed students T test.

MN1 expression confers resistance to doxorubicin in human AML cells. In order to determine if the findings in our murine model hold true for human AML cells, we partially infected the human cell line OCI-AML3 with our MN1-expressing vector. We chose OCI-AML3 cells because, as opposed to most AML cell lines, they have an intact p53 response. Consistent with our mouse model, in repeated experiments MN1 cells were selected for by increasing amounts of doxorubicin (Figure 7A). The control vector showed no enrichment under any of the conditions assayed. To see if this result would be reproducible in cell line with mutated p53 we infected Molm-13 cells with the MN1-expressing vector. Interestingly, a more modest but still significant enrichment of MN1-expressing cells was seen when cells were exposed to doxorubicin (Figure 7B). No enrichment of MN1 was observed when either cell line was treated with cytarabine. This may reflect differences between human and mouse responses to MN1, or cell line-specific responses. Overall, these data suggest that MN1 confers resistance to doxorubicin in human AML cells.

## Discussion

AML is aggressive, genetically heterogenous malignancy with poor outcomes. Patients can be divided into different prognostic groups on the basis of cytogenetics [3], [4]. There is an established link between cytogenetics and response to chemotherapy as remission rates differ significantly from one cytogenetic risk group to another [33]. In addition to gross chromosomal abnormalities, overexpression or mutation of certain genes has also been implicated in prognosis for patients with AML [34]. One such gene is the transcriptional co-activator MN1. MN1 expression levels inversely correlate with prognosis in both younger and older AML patients [9], [10], [11]. MN1 overexpression can cooperate in the generation of AML [17], [18], [35] and can generate AML as a single oncogene in mice [16]. High expression of MN1 predicts resistance to ATRA in elderly patients [16]; however, the effect of MN1 overexpression on response to standard chemotherapy and how this contributes to adverse prognosis is not well understood. To investigate the effects of MN1 overexpression, we used a genetically defined murine AML model and human AML cell lines.

MN1 overexpression in MLL-ENL-driven murine AML cells led to cytokine independence and conferred a significantly worse survival. MLL-ENL and Flt3 ITD murine AML cells overexpressing MN1 led to a statistically significant shortening of survival in syngeneic recipients when compared to GFP-only cells in both treated and control groups. Second, MN1 conferred resistance to chemotherapy *in vitro* and *in vivo*. Third, MN1 did not decrease the amount of DNA damage induced by doxorubicin, but did suppress apoptosis. Fourth, MN1 overexpression resulted in suppression of p53 and BIM induction following treatment. The levels of p53 in MN1 expressing cells could be increased with the MDM2 antagonist nutlin 3 and concurrent treatment with nutlin 3 and doxorubicin resulted in increased cell death. Finally, MN1 overexpression in a human AML cell with an intact p53 response led to resistance to doxorubicin while a much more modest resistance was seen in a human AML cell line with mutated p53.

These data suggest that MN1 overexpression adversely affects prognosis by suppressing proapoptotic signaling, either via loss of survival signals or DNA damage. Cytokine withdrawal normally leads to apoptotic cell death that can involve p53 [36], [37]. MN1 overexpression compensates for this loss of prosurvival signaling, and is consistent with previously observed growth-stimulatory effects of high MN1 expression in AML cells [18]. This loss of cytokine dependence may also contribute to the shortened survival in mice, as leukemic blasts would be less dependent on the cytokine milieu in the marrow for growth. This cytokine-independent growth was not seen with the control GFP vector, which demonstrates that functional MN1 was expressed in our system.

The increased therapeutic resistance is likely the result of disconnecting the DNA damage response (DDR) from the ability to commit to apoptosis. γH2AX foci formation and CHK-1 phosphorylation were both preserved in response to doxorubicin, arguing that the initial DNA damage-sensing ability is still present in MN1 expressing cells, since as they represent the initial events in the DDR [38]. However, downstream signaling was impaired, as demonstrated by the lack of p53 induction following DNA damage and loss of apoptosis. Altered DDR has been reported in AML, with increasing DDR activation seen in pre-leukemic states (i.e., Myelodysplastic syndrome) and then suppression in AML [39]. In a recent study that assessed DDR and apoptosis patterns in primary AML samples, a group of patient samples displayed an intact DDR, as evidenced by induction of CHK2 phosphorylation but no apoptosis. However, no patients in that cohort showed a clinical response to treatment, implying that a lack of effective induction of apoptosis through DDR leads to clinical failures and worse prognosis [40]. Increased MN1 expression may represent a means by which some AML samples uncouple efficient DDR and apoptosis.

In addition to loss of p53 induction in response to DNA damage, we also observed attenuated BIM message and protein induction. BIM is transcriptionally regulated primarily by the forkhead transcription factor, FOXO3a. In response to activation of prosurvival signaling, this molecule is phosphorylated, exported to the cytosol, and either sequestered by 14-3-3 proteins or degraded following ubiquitination [41]. Intriguingly, FOXO3a is also involved in DDR. It physically interacts with ATM and γH2AX at sites of DNA damage and influences the G2-M cell cycle arrest following irradiation [40]. FOXO3a dysregulation has also been implicated in AML, as samples with elevated FOXO3a message levels or hyperphosphorylated FOXO3a protein were associated with a worse prognosis [42], [43]. Additionally, the hypomethylating agents azacitidine and decitabine may act in part by reversing FOXO3a phosphorylation [44]. We speculate that MN1 overexpression may act in part by deregulating FOXO3a activity, leading to impaired DDR and inability to effectively induce BIM after treatment with DNA-damaging agents.

These data suggest that MN1-expressing cells suppress p53 and BIM, leading to resistance to apoptosis, and provide a mechanism for the effect of MN1 on prognosis. The concurrent use of p53 stabilizing agents with standard chemotherapy may lead to improved outcomes for patients with high MN1 expression and should be tested in clinical trials.

## Supporting Information

Figure S1 A) Schematic overview of the generation of the M1p5, MF3 and MFL2 cell lines. C57BL/6 fetal liver cells isolated at E13.5–E15 were (co)transduced with MLL-ENL-expressing retroviruses and injected into lethally irradiated recipients. B) Flow cytometry histogram. Partially infected population of MLL-ENL cells were cultured with and without cytokines (murine SCF, murine iL6, and murine iL3) for indicated durations. Shown are histograms for GFP expression of the viable cell population from a representative experiment.
(TIF)

Figure S2 MN1 expression accelerates AML onset. Kaplan-Meier survival curves. MFL2 cells infected with GFP-or MN1-expressing vector were injected into Ly5.1+ C57Bl/6 mice. Mice were then followed for survival. Curves were compared using the log rank test.
(TIF)

Figure S3 MN1 expression confers resistance to Flt3 expressing AML. A) Flow cytometry histograms. Partially infected population of MF3 cells were exposed to increasing amounts of doxorubicin as indicated for 72 hours and analyzed for GFP expression. Shown are histograms for GFP expression of the viable cell population from a representative experiment. B) Competition assays. Partially infected MF3 or MFL2 cells were exposed to the indicated treatment for 72 hours. GFP-positive percentage in the viable population was determined and normalized to untreated controls. Shown is the average of three independent experiments each done in triplicate. Error bars represent the standard error of measurement. P value was calculated using a 2 tailed students T test. C) Viability assays. Purified populations of MF3 cells with GFP or MN1 were exposed to the indicated amounts of doxorubicin or cytarabine (Ara-C) for 72 hours and viability determined. Shown is the average of three independent experiments each done in triplicate. Error bars represent the standard error of measurement. P value was calculated using a 2 tailed students T test.
(TIF)

Figure S4 Schema of *in vivo* competition assays. A) Schema of *in vivo* competition assays. B) Gating strategy employed on isolated marrow cells from mice treated as in A.
(TIF)

Figure S5 MN1 expression decreases apoptosis. A) Annexin V and propidium iodide staining of M1p5 cells expressing GFP or MN1. Cells were exposed to the indicated treatments for 48 hours, stained with PI and annexin V and analyzed by flow cytometry. B) Annexin V and propidium iodide staining of MF3 cells expressing GFP or MN1. Cells were treated and analyzed as in A.
(TIF)

Figure S6 MN1 expression suppresses p53 response. A) Nutlin 3 titration. MF3 cells were exposed to increasing amounts of nutlin 3 for 72 hours and viability determined. Shown is the average of three independent experiments each done in triplicate. Error bars represent the standard error of measurement. P value was calculated using a 2 tailed students T test. B) Western blots. MF3 cells were exposed to the indicated amount of Nutlin-3 for 6 hours and blotted for p53. Actin served as a loading control.
(TIF)

Figure S7 Nutlin 3 increases doxorubicin sensitivity to MN1 expressing cells. Doxorubicin titration in the presence and absence of Nutlin 3. M1p5 cells expressing GFP were exposed to a titration of doxorubicin. M1p5 cells expressing MN1 were exposed to the same titration of doxorubicin in the absence and presence of 5 µM nutlin 3.
(TIF)
