# Supplementary figures and images for: Overexpression of MN1 Confers Resistance to Chemotherapy, Accelerates Leukemia Onset, and Suppresses p53 and Bim Induction
Source: PLoS One. 2012 Aug 14;7(8):e43185. doi: 10.1371/journal.pone.0043185 (PMC3419213; doi:10.1371/journal.pone.0043185)

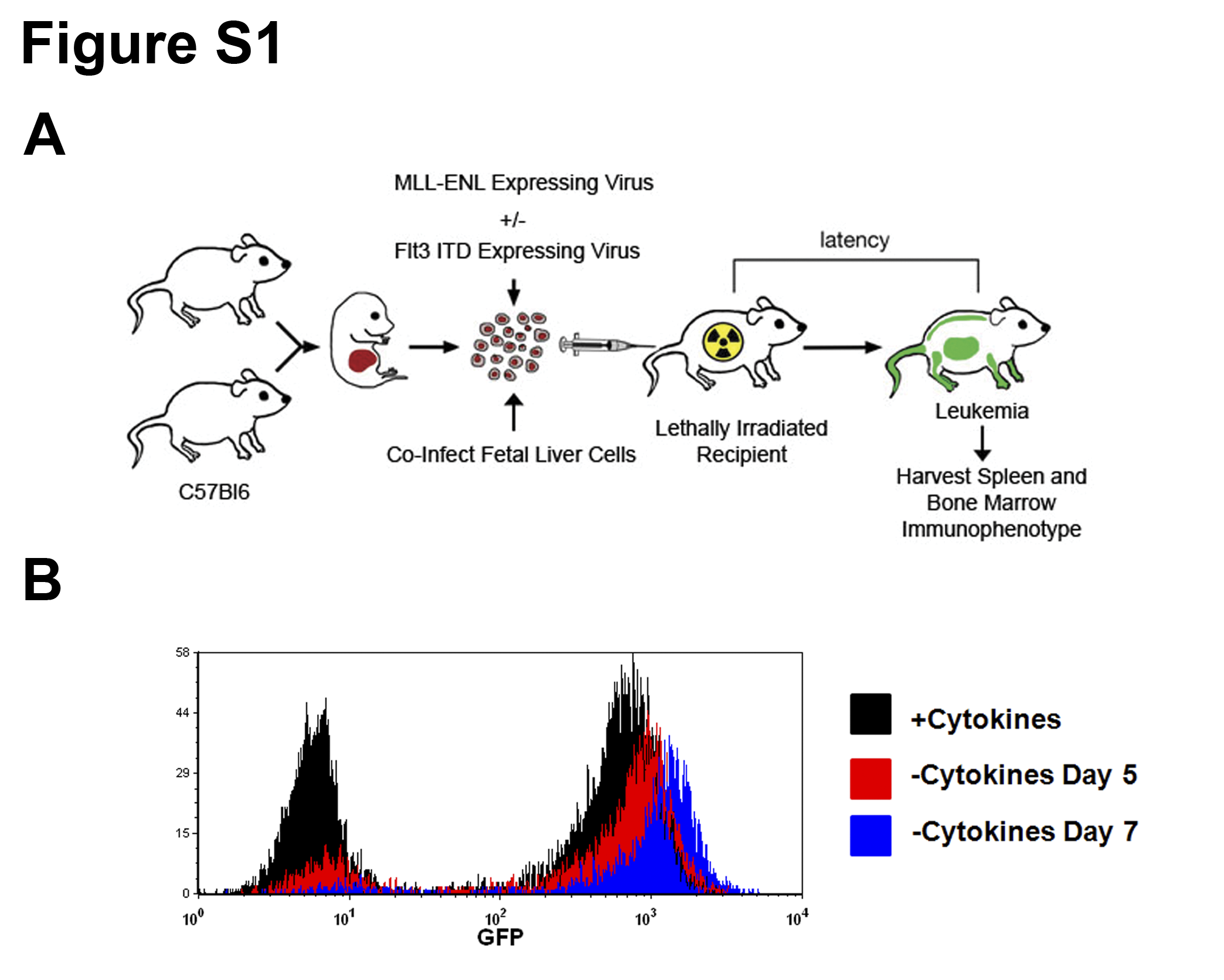

Supplement: Figure S1 — A) Schematic overview of the generation of the M1p5, MF3 and MFL2 cell lines. C57BL/6 fetal liver cells isolated at E13.5–E15 were (co)transduced with MLL-ENL-expressing retroviruses and injected into lethally irradiated recipients. B) Flow cytometry histogram. Partially infected population of MLL-ENL cells were cultured with and without cytokines (murine SCF, murine iL6, and murine iL3) for indicated durations. Shown are histograms for GFP expression of the viable cell population from a representative experiment. (TIF) [file pone.0043185.s001.tif]

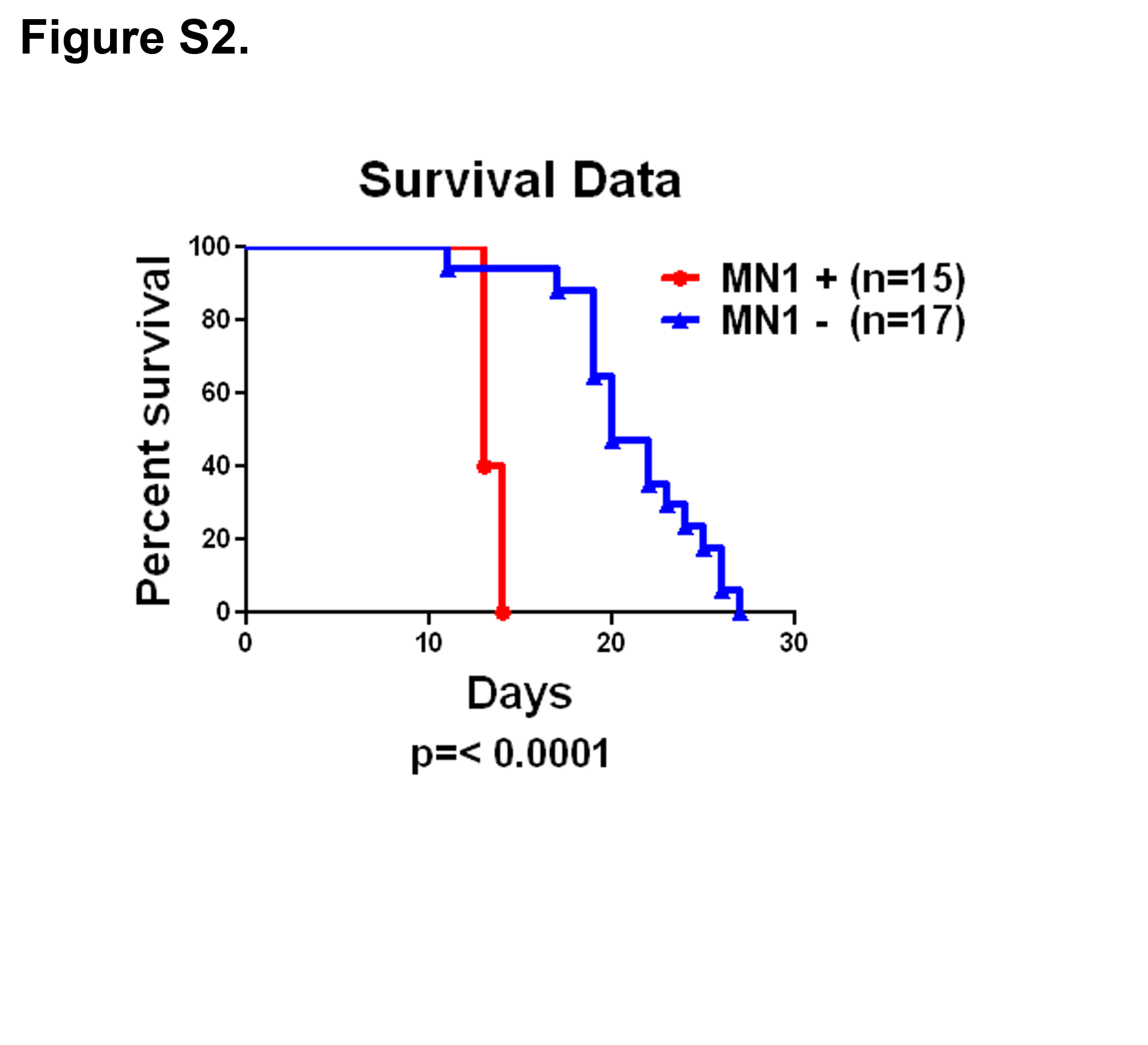

Supplement: Figure S2 — MN1 expression accelerates AML onset. Kaplan-Meier survival curves. MFL2 cells infected with GFP-or MN1-expressing vector were injected into Ly5.1+ C57Bl/6 mice. Mice were then followed for survival. Curves were compared using the log rank test. (TIF) [file pone.0043185.s002.tif]

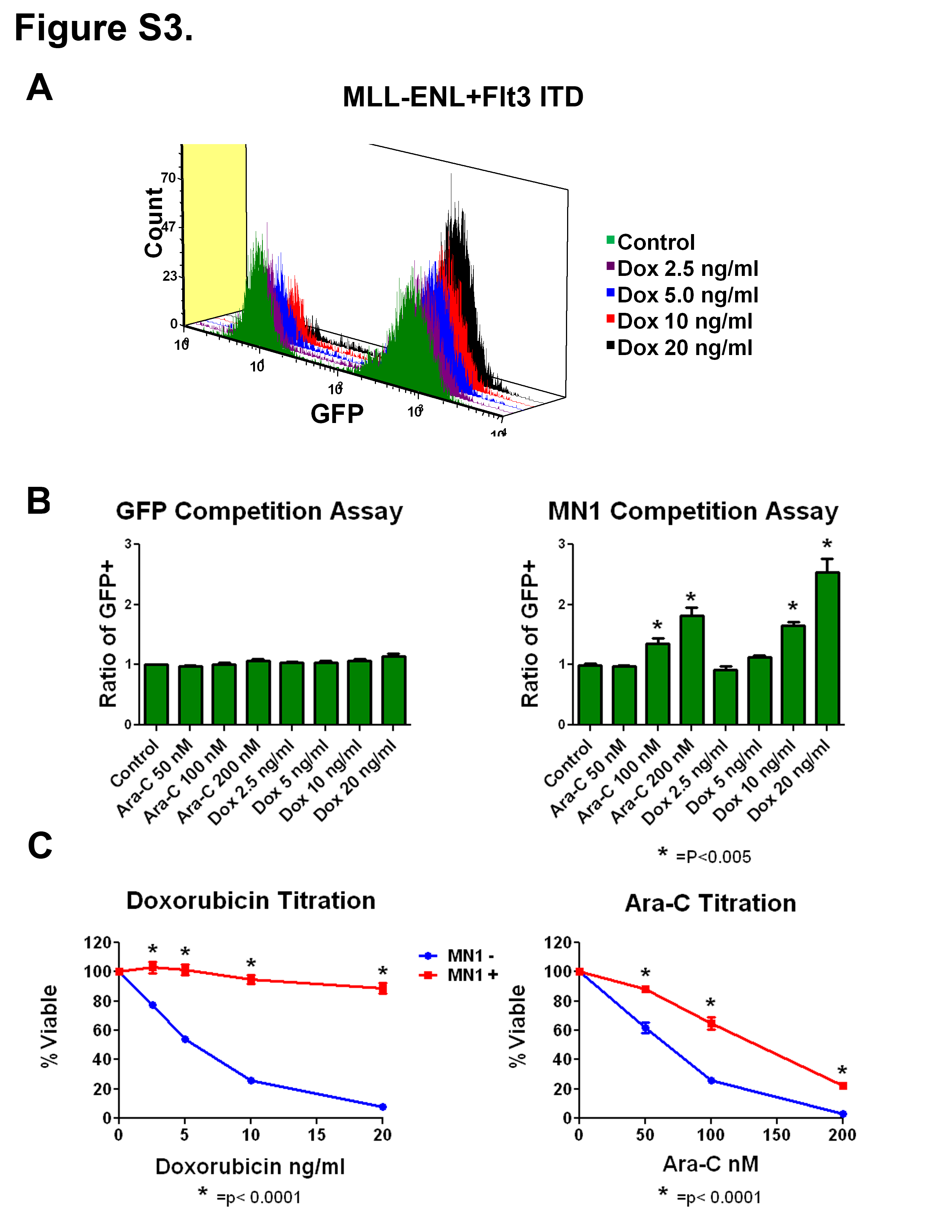

Supplement: Figure S3 — MN1 expression confers resistance to Flt3 expressing AML. A) Flow cytometry histograms. Partially infected population of MF3 cells were exposed to increasing amounts of doxorubicin as indicated for 72 hours and analyzed for GFP expression. Shown are histograms for GFP expression of the viable cell population from a representative experiment. B) Competition assays. Partially infected MF3 or MFL2 cells were exposed to the indicated treatment for 72 hours. GFP-positive percentage in the viable population was determined and normalized to untreated controls. Shown is the average of three independent experiments each done in triplicate. Error bars represent the standard error of measurement. P value was calculated using a 2 tailed students T test. C) Viability assays. Purified populations of MF3 cells with GFP or MN1 were exposed to the indicated amounts of doxorubicin or cytarabine (Ara-C) for 72 hours and viability determined. Shown is the average of three independent experiments each done in triplicate. Error bars represent the standard error of measurement. P value was calculated using a 2 tailed students T test. (TIF) [file pone.0043185.s003.tif]

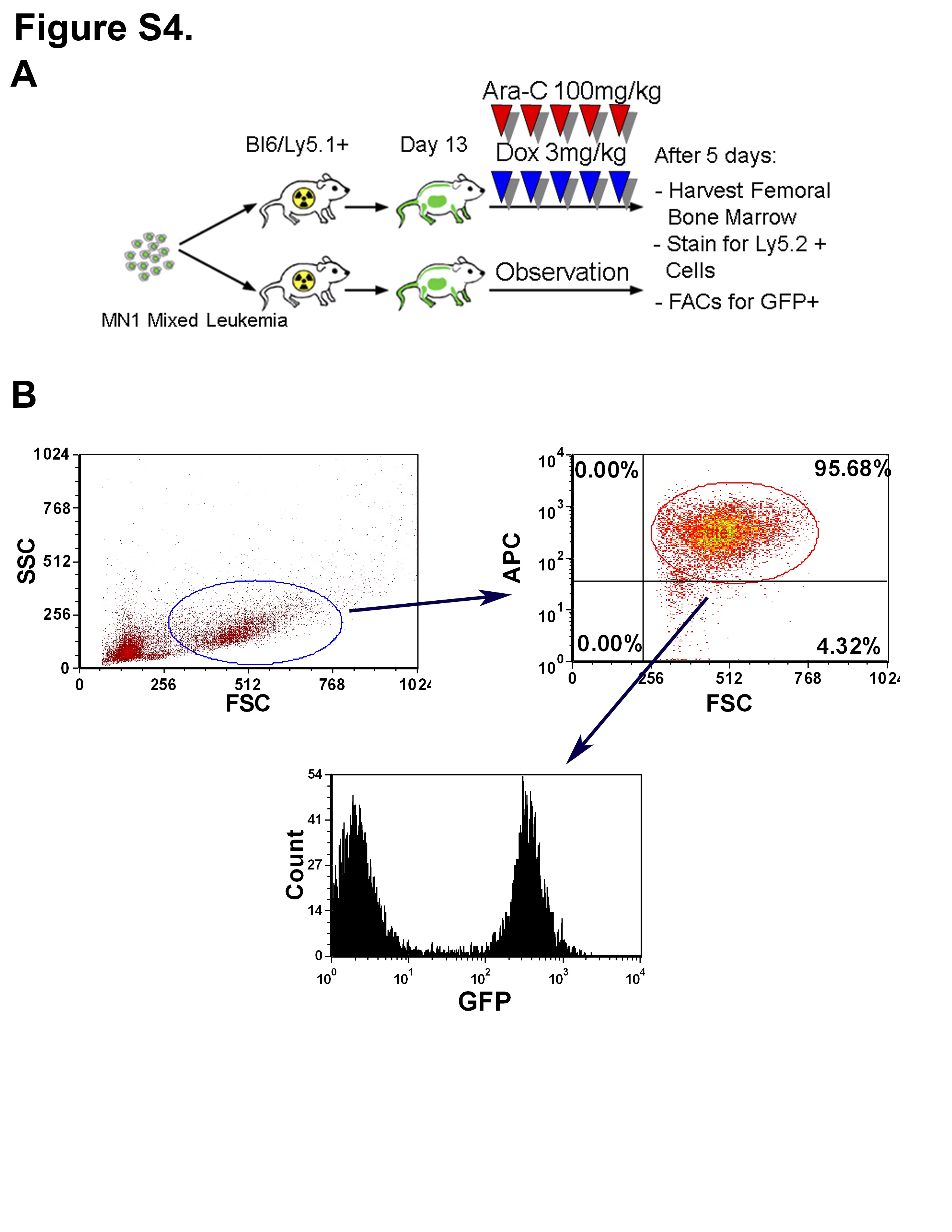

Supplement: Figure S4 — Schema of in vivo competition assays. A) Schema of in vivo competition assays. B) Gating strategy employed on isolated marrow cells from mice treated as in A. (TIF) [file pone.0043185.s004.tif]

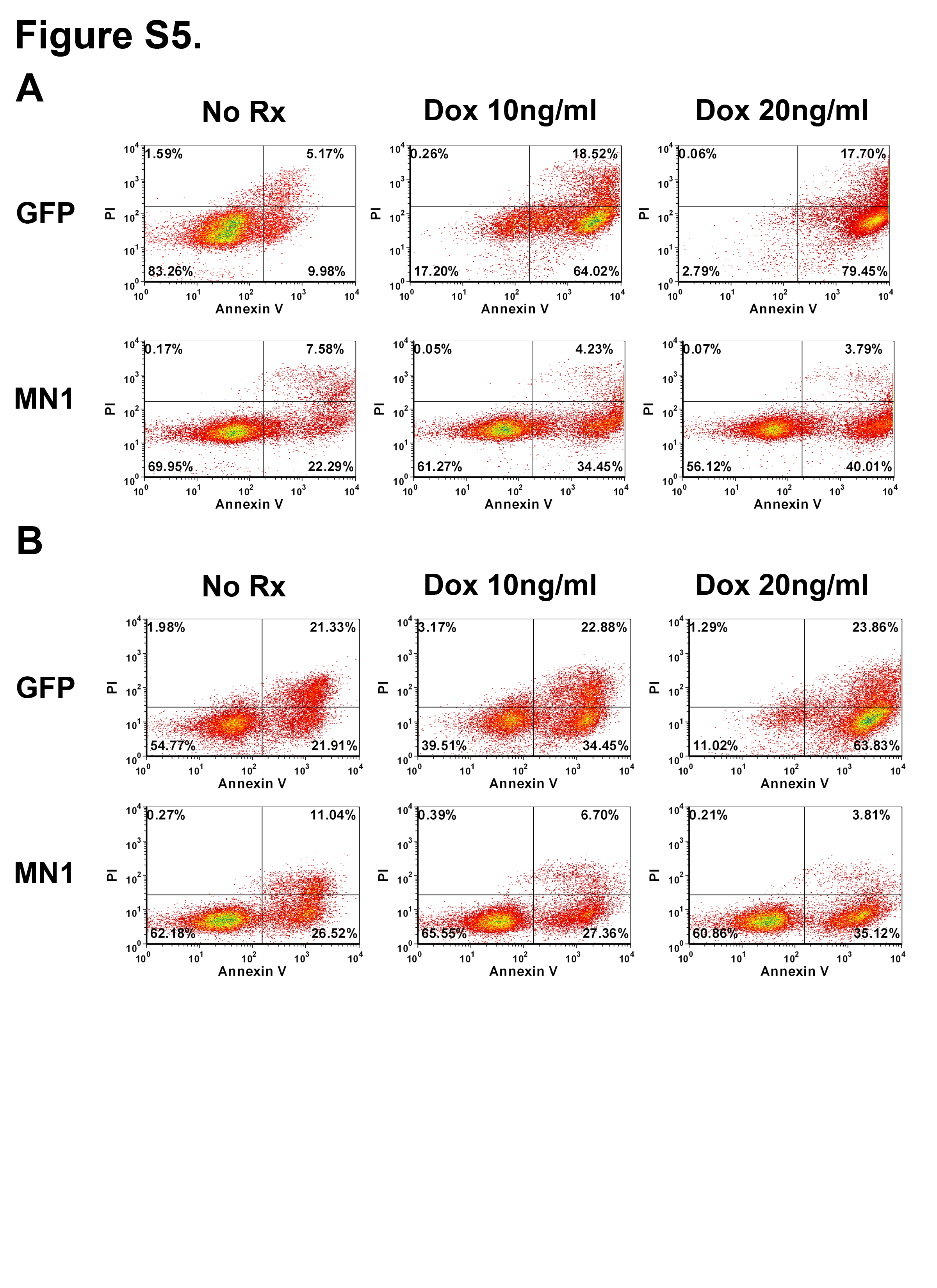

Supplement: Figure S5 — MN1 expression decreases apoptosis. A) Annexin V and propidium iodide staining of M1p5 cells expressing GFP or MN1. Cells were exposed to the indicated treatments for 48 hours, stained with PI and annexin V and analyzed by flow cytometry. B) Annexin V and propidium iodide staining of MF3 cells expressing GFP or MN1. Cells were treated and analyzed as in A. (TIF) [file pone.0043185.s005.tif]

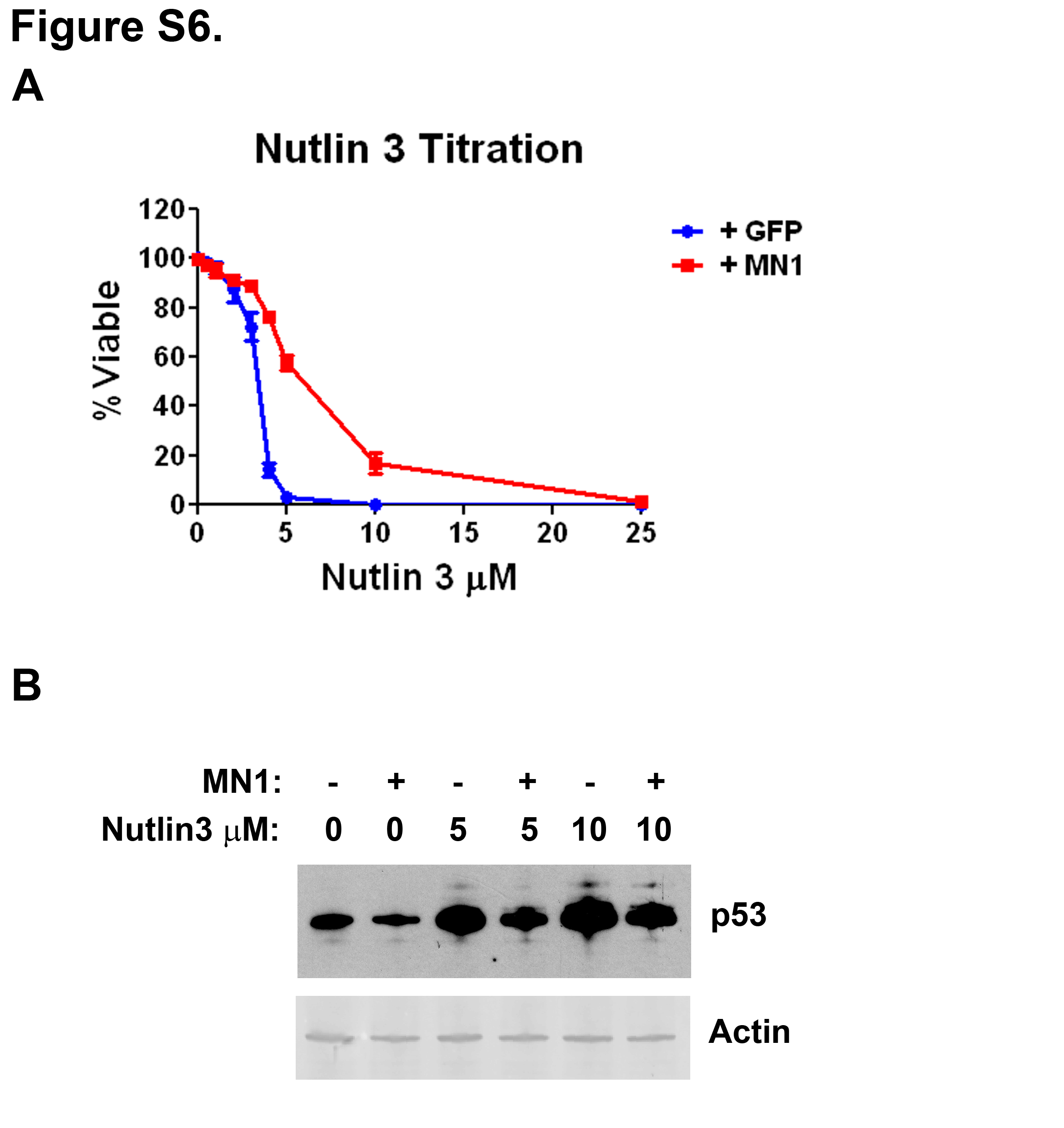

Supplement: Figure S6 — MN1 expression suppresses p53 response. A) Nutlin 3 titration. MF3 cells were exposed to increasing amounts of nutlin 3 for 72 hours and viability determined. Shown is the average of three independent experiments each done in triplicate. Error bars represent the standard error of measurement. P value was calculated using a 2 tailed students T test. B) Western blots. MF3 cells were exposed to the indicated amount of Nutlin-3 for 6 hours and blotted for p53. Actin served as a loading control. (TIF) [file pone.0043185.s006.tif]

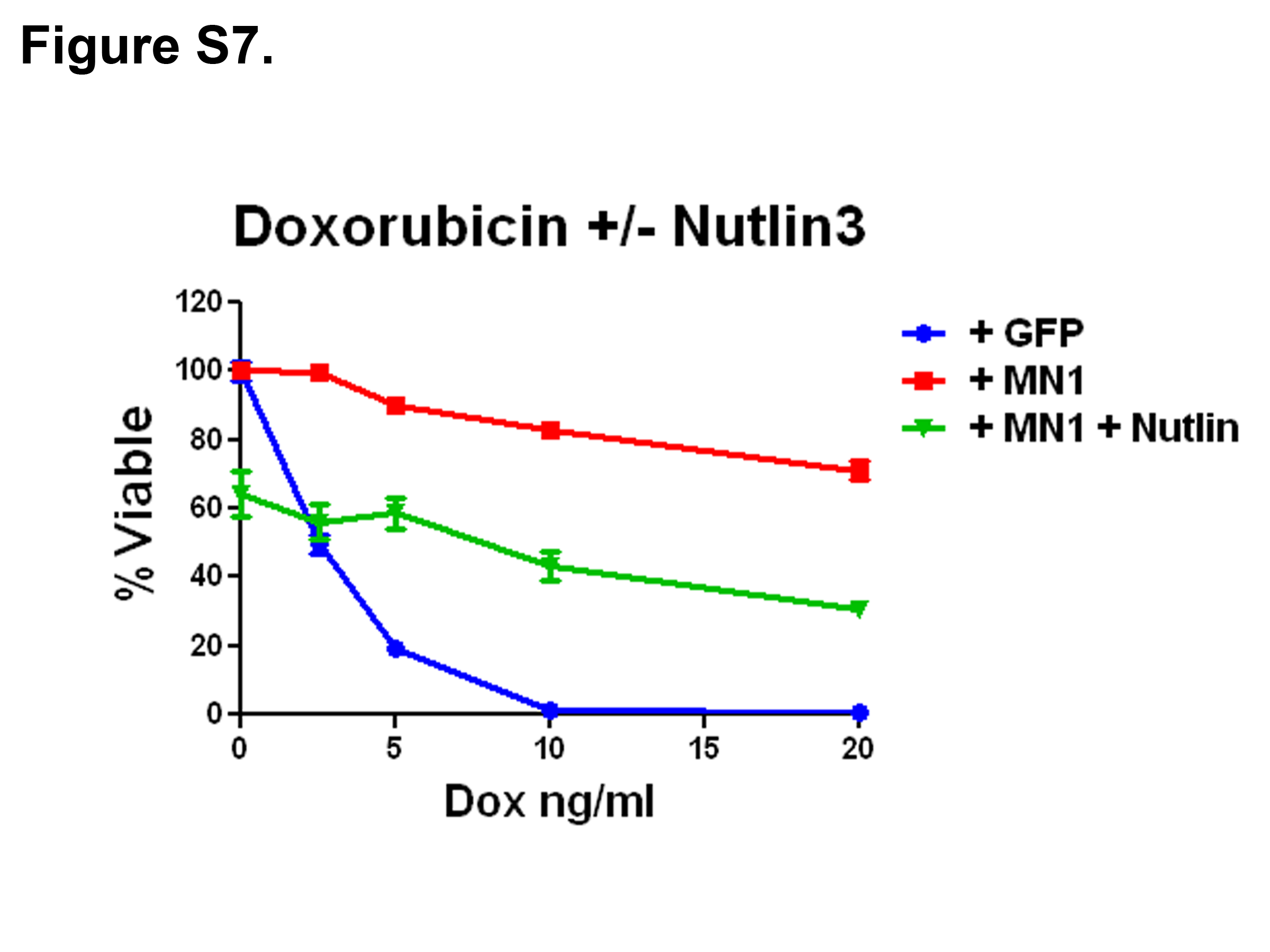

Supplement: Figure S7 — Nutlin 3 increases doxorubicin sensitivity to MN1 expressing cells. Doxorubicin titration in the presence and absence of Nutlin 3. M1p5 cells expressing GFP were exposed to a titration of doxorubicin. M1p5 cells expressing MN1 were exposed to the same titration of doxorubicin in the absence and presence of 5 µM nutlin 3. (TIF) [file pone.0043185.s007.tif]
